# Generating traffic flow and speed regional model data using internet GPS vehicle records

**DOI:** 10.1016/j.mex.2019.08.018

**Published:** 2019-09-10

**Authors:** Sergio Ibarra-Espinosa, Rita Ynoue, Mariana Giannotti, Karl Ropkins, Edmilson Dias de Freitas

**Affiliations:** a Departamento de Ciências Atmosféricas, Instituto de Astronomia, Geofísica e Ciências Atmosféricas, Universidade de São Paulo, Brazil; b Key Laboratory of Wetland Ecology and Environment, Northeast Institute of Geography and Agroecology, Chinese Academy of Sciences, Changchun 130102, China; c Laboratory of Geoprocessing, Escola Politécnica (Poli), Universidade de São Paulo, Brazil; d Institute for Transport Studies, University of Leeds, United Kingdom

**Keywords:** Traffic flow generation based on GPS recordings, GPS, Vehicles, Spatial bias, Internet, Data, Brazil

## Abstract

Nowadays, many smart-phones and vehicles are equipped with Global Position System (GPS) for tracking and navigation purposes, providing an opportunity to derive highly representative local vehicular flow and estimate vehicular emissions information. Here, we report and discuss methods used to handle large volumes of such activity data, namely 124 million GPS recordings from the web page Maplink.com.br, extract high spatial resolution vehicular flow information for a vast area in South-east Brazil, and correct for bias using traffic counts observations for the same area. The method consists in **filter** speed and accelerations, **assign** buffers to the road network, **aggregate** speed by street, **fill** missing number of lanes, generate traffic **flow**. Methods presented here were used to inform traffic-related air quality modelling and used as part of local air pollution management activities but are also amenable to any work that would be enhanced by more locally representative or time-resolved inputs for traffic flow, e.g. traffic network management, and demand modelling.

• 124 million GPS observations from electronic devices were used to generate traffic flow.
• Spatial bias was investigated and accounted for using independent local traffic count data.
• Traffic count rescaled GPS traffic flow provide a robust description of spatial and quantitative traffic patterns.

## Graphical abstract

**Figure:**
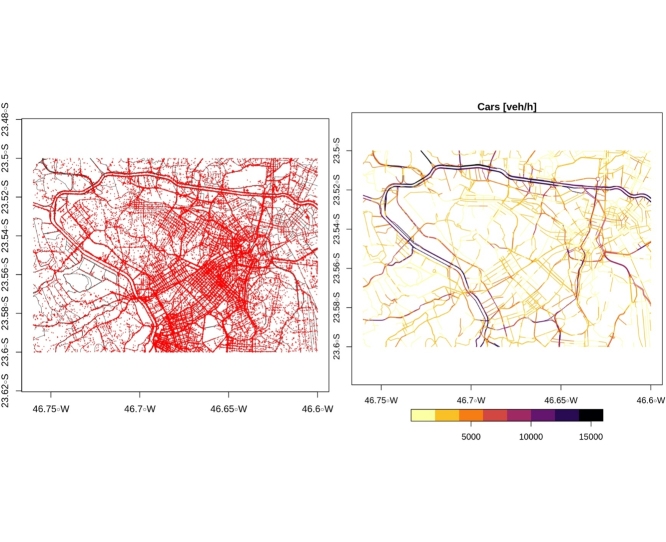

**Specifications Table**

**Table:** 

Subject Area:	Environmental Science
More specific subject area:	Traffic flow information extraction using (1) primary extraction of flow information from large GPS datasets, and (2) correction for local bias using independent local vehicle counts.
Method name:	Traffic flow generation based on GPS recordings
Name and reference of original method:	Non-applicable.
Resource availability:	*R:*https://cran.r-project.org/ *sf:*https://github.com/r-spatial/sf *data.table:*https://CRAN.R-project.org/package=data.table *Traffic counts:*https://doi.org/10.17632/rz2cymv6c2.1

## Method details

**Fig. 1:**
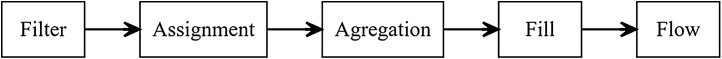
Workflow used in this work.

Nowadays many smart-phones and vehicles incorporate Global Position Systems (GPS) for many uses. Although most widely used as a navigation tool, data collected by these systems are available for use in an increasing number of applications, including accident investigation, georeferencing, and real-time traffic management and demand modelling (see e.g. [[1], [2], [3]]). Here, we specifically present methods to extract and bias-correct regional-scale traffic flow information derived from a high-volume vehicular GPS data source. In this case that information is used as an input to VEIN, a bottom-up vehicle emissions model freely available as an R package [4], which is being actively used as part of local air quality management and research activities, but the approach and methods presented here are also potentially amenable to any application that would benefit from representative, time-resolved information on traffic flow and other related properties of vehicle activity. Therefore, in this study we applied the workflow shown on Fig. 1.

The steps used in our method are:

1 **Filter**: Calculate the distance and time for consecutive observations of the same vehicle, then calculate the speed and accelerations and then filter the data.
2 **Assignment**: Transform the data in spatial points, create a buffer on each point and intersect them with the Open Street Map road network.
3 **Aggregation**: Aggregate speed measurements by each street.
4 **Fill**: Some Open street Map roads do not have number of lanes identified, therefore these are filled with average by type of street.
5 **Flow**: Associate the speeds measured in Assignment with an external data-base of simultaneous speed and traffic volume recordings aggregated by type of street and number of lanes to generate traffic flow estimate.

Below, a more detailed description on each step.

### Filter

GPS vehicle tracking data is typically logged as a continuous registry of the time-stamped geographical positions. The frequency of the registration varies depending on the GPS characteristics of each vehicle but is approximately 30 s to 1 min in Brazil.

The GPS data used in this study was obtained from Maplink (http://transito.maplink.global/), one of the companies that provides data to Google. Maplink obtains anonymous vehicle GPS data from several other companies to provide broad coverage of different vehicle types. The GPS data was stored online, and we extracted the data for the study area coordinates using the Google online tool Biq Query (https://cloud.google.com/bigquery/). The data is composed of GPS positions of more than 124 million GPS positions from 211,247 vehicles for the study area and includes the following 5 columns: "Vehicle", an anonymous id for each vehicle; "Type", a vehicle type descriptor, including the designations Cars, Taxi, Trucks and Undefined; "Collect_time", a time-stamp with the format Year-month-day Hour-Minute-Seconds (eg: "2014-10-05 00:00:00") in UTC; and, "Lat" and "Long" respectively indicating the geographical coordinates in the WGS 84 spatial reference system. The extracted time period used here to demonstrate traffic flow extraction methods was "2014-10-05 00:00:00" to "2014-10-11 00:00:00", and covered 6 days, from Saturday October 4th at 21:00 of 2014 to Friday 10th at 21:00 of 2014 in Local Time (LT).

**Fig. 2:**
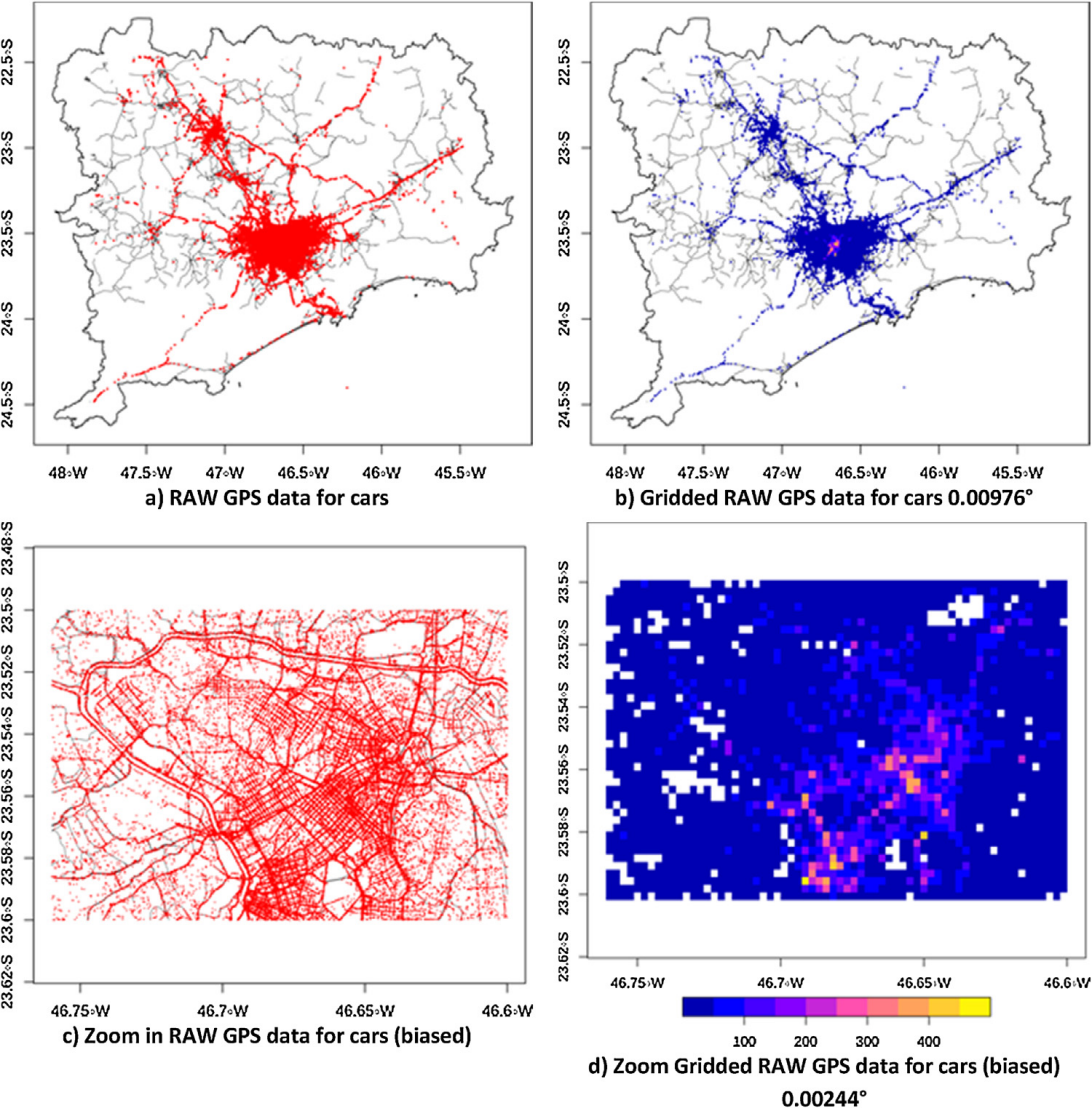
(a) Raw GPS records of Cars, (b) gridded raw GPS records of Cars with spacing of 0.00976°, c) zoom at the GPS records of Cars and d) zoom at the gridded GPS of cars with grid spacing of 0.00244°.

The associated data request generated 50 Comma Separated Value (CSV) text files of raw GPS data. Each file contains information for the 145 h and had to be merged and cleaned-up before use. As part of that process we developed quality control strategies for the identification and exclusion of suspect records, and data-subsets not suitable for traffic flow analysis. We calculated the great circle distance between two consecutive readings of each vehicle to calculate its speed and acceleration. The quality control included the following rules: no vehicle could have speeds higher than 110 km h^−1^ and acceleration higher than 129,600 km h^-2^, as suggested by Nhan et al, (2016). Also, that the same vehicle must be tracked at least in 5 times in each CSV file. At this point, the total number of observations was 124,198,931 (more than 124 million), with 11,497,745 observations of 10,239 Passenger Cars (Light Duty Vehicles – LDV), 46,798,568 observations of 21,514 Taxi, 419,596 observations of 30,833 Trucks (Heavy Duty Vehicles – HDV), and 61,783,022 observations of 148,661 Undefined vehicles. As the data is too numerous to plot in full, we are showing slices of the data from Passenger Cars in Fig. 2

Generating traffic flow for emissions inventories from massive GPS records is, to our knowledge, a novel approach and it requires caution. One important aspect is the frequency of the observations. Ideally, the frequency of two consecutive observations from the same vehicle would be about 1 Hz (one measurement per second). However, the average time between our records is 9,154.900 s for Cars and 38,345 s for Trucks. This means that on average, there is 2.54 h between two consecutive records of the same Car and 10 h between records of the same Truck. Therefore, we filtered the data to include only consecutive observations of higher frequency. This step obviously reduces the number of observations. Therefore, this step is an unavoidable compromise between a high frequency of consecutive observations and a minimum set of observations by each street. We explored several frequencies until we achieved a reasonable street coverage. A maximum frequency of 5 min resulted in an average of 2.21 consecutive readings and barely 50 streets. A maximum frequency of 10 min resulted in an average of 4.27 min resulted in approximately 2000 streets with records. Finally, a maximum frequency of 29 min resulted in an average of 11.57 min and approximately 5,000–6,000 streets with observations per hour. Although at this stage street numbers were still lower than we would have liked, we decided to use an average frequency of 11 min because larger maximum frequencies increased the likelihood of including more disperse data less representative of particular locations, local road types and driving conditions. The consequence of this decision is that speed calculation is more reliable for observations with higher frequency. The percentiles for the data with 11 min of frequency for Cars were P25 = 240 s, P50 = 600 s, P75 = 1095 s and P100 = 1799 s. We believe that future studies could further improve on this approach by interpolating GPS locations at a higher frequency using for instance, the shortest path approach. However, accessing a GPS data-set with high frequency of observations is clearly preferred, where possible. Despite limitations of data, we believe that this study is a significant step forward.

In order to assess the spatial distribution of the resulting GPS recordings, we plot Cars data from one of the 50 CSV files in Fig. 2. Each one of these files includes registers for all hours. GPS recordings presents a good spatial distribution as demonstrated by comparing Fig. 2a and b. One of our earliest findings was that most of recordings were for non-residential streets. One possible reason is that vehicles move quickly from a residential street to another street types (e.g. when going to or coming from work) but more often travel along routes which are predominantly of other street-types. We therefore excluded this type of street from the analyses. Another early finding was the presence of spatial bias on GPS recordings as shown on Fig. 2b, c and d with a higher density of GPS recordings on the west side of São Paulo city, possibly related with the neighborhoods with higher income in this region.

### Assignment

#### Data treatment

We used the R package **data.table** [5] for handling this data because it provides fast analysis with few computational costs. As the Taxis, Cars, Trucks and Undefined share a common space on each street, we use the information of all vehicles to calculate the speed for each individual vehicle. We used a personal computer (laptop DELL Intel Core i7-5500U CPU 3 GHz, 16 Gb RAM) in this study. The objective was characterizing the vehicle speed by type of vehicle, for all vehicles, on each road and hour of the study period.

As traffic data are points and we must assign them to the road network, we first converted the traffic data to spatial features with geometry points and projected the data to the system of coordinates 31983 SIRGAS 2000 / UTM zone 23 South using the R package **sf** [6]. In order to avoid impractically high RAM usage, we divided the data by hour and then created buffers with 10 m distance to have one polygon to each traffic recording position, which means that we were also adding vehicular speed to each street. Then we made an intersection between the polygons and road network so that each street had a speed for each vehicle. Finally, we aggregated the speeds by each street calculating mean, median, quartile .75, .85, .95 and max speeds by type of vehicle and hour. We also generated another data base with the same statistics of speed but now only for each street and hour.

Taxis were the most common vehicle type in the local fleet. Nowadays, almost every Taxi in the local fleet has a GPS, and most of the records is available. This means that although the total number of Taxis in circulation is lower than the total number of Cars, Taxis GPS coverage is much higher. To ensure that the calculation of the speed was correct we calculated the average speed by type of street for an early morning hour. In Table 1 we show the mean speed by type of street for Cars, Taxi, Trucks, and the Max Speed for Taxi and for all type of vehicles. The average speeds on most road types was similar, except for motorways, where obviously speeds were much higher.

**Table 1:** Speeds on Monday 2014-10-06 00:00 by type of street (km h^−1^).

Type of street	Cars_mean_	Taxi_mean_	Truck_mean_	Taxi_max_	Trucks_max_	All_mean_
Motorway	73.98	40.40	55.09	51.45	57.14	65.63
Motorway_link	–	36.99	56.96	41.53	57.83	52.44
Trunk	14.80	22.73	43.49	26.39	43.50	37.84
Trunk_link	–	22.09	–	23.26	–	34.99
Primary	16.09	16.38	44.12	23.26	45.22	27.12
Primary_link	22.00	18.44	–	21.30	–	28.04
Secondary	7.65	14.69	50.80	19.21	60.61	22.24
Secondary_link	0.06	18.64	–	21.00	–	25.39
Tertiary	4.17	12.59	42.32	16.54	43.31	19.59
Tertiary_link	–	16.00	56.65	16.54	72.20	24.49
Mean	10.31	17.76	54.19	23.46	55.96	31.37
n	64	5679	147	5679	147	9,119

### Aggregation

Ideally, in order to represent Cars, we should consider only records of this vehicle type that have an average speed of 74 km h^-1^ on motorways and lower speeds on other type of streets. However, the number of such records was very small (64/56966 streets). Therefore, we used the speed of Taxis, for which coverage was much higher (5679 streets) as a proxy of Cars traffic flow. This was not ideal because the mean and maximum speeds of Taxis were higher than Cars on motorways, and lower on other street types, which could be related to the different behavior of Taxis in comparison with Cars. Trucks were found to travel at higher speeds than Cars and Taxis on all types of streets except motorways, perhaps in part reflecting hours of operation, active congestion avoidance by commercial fleet managers and greater GPS coverage on interurban roads. Lastly, the mean speed of all vehicles (aggregating Cars, Taxi, Trucks and Undefined) were within expected ranges.

In order to produce robust traffic flows despite the limitations of the data, we decided to investigate the use of both the average maximum speed of taxis and average speed for all vehicles as proxies for traffic flow of Cars. In the case speed of Trucks, we took a similar approach, considering first the average maximum speed of Trucks and, when there was no data available, the average maximum speeds of all vehicles, which seemed appropriate given that Truck and all Vehicle speeds were often highly similar. The procedure for generating traffic flows is explained in following sections.

**Fig. 3:**
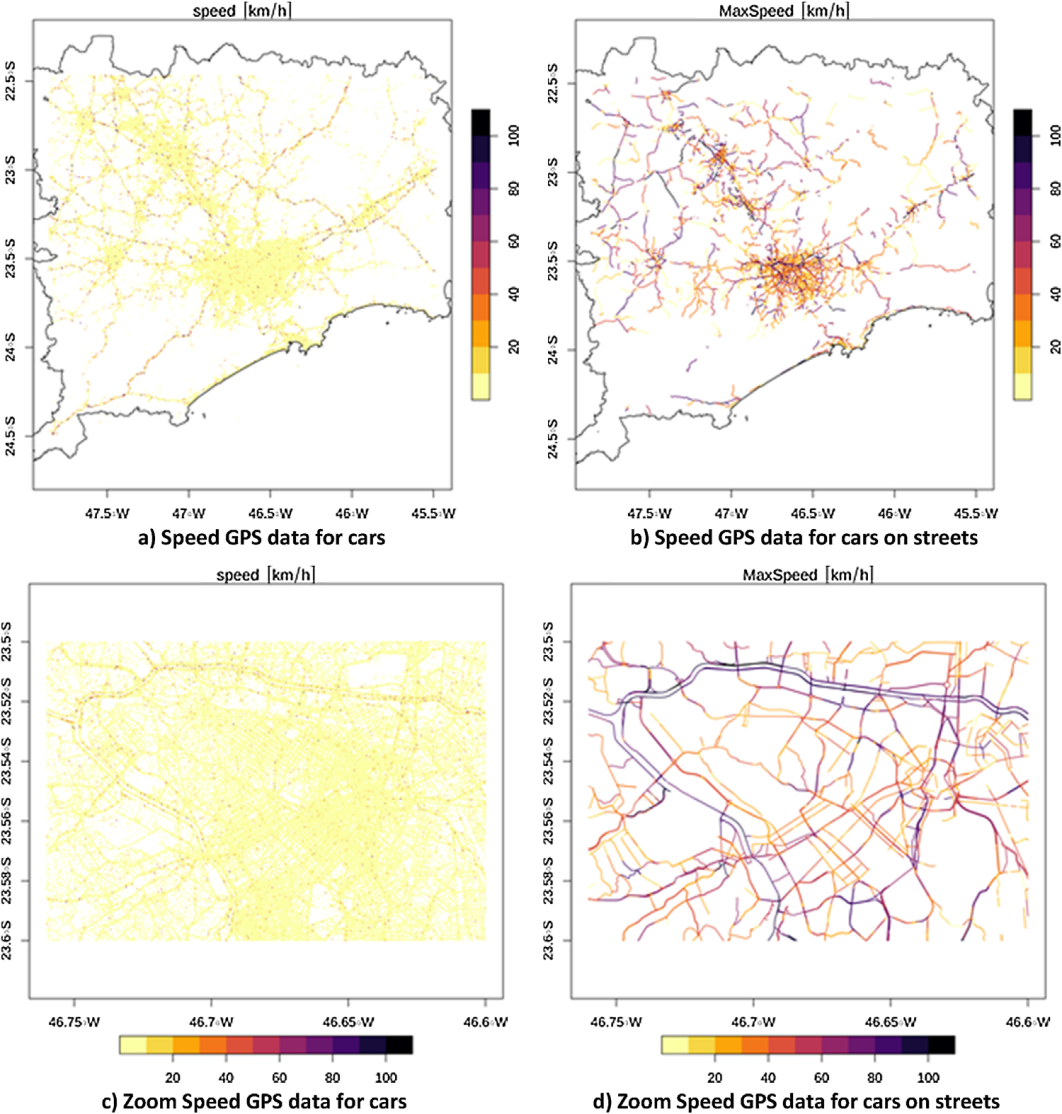
a) Calculated speed (km h^−1^) of Cars, b) calculated max Speed (km h^−1^) on streets, c) zoom at calculated speed (km h^−1^) of Cars, d) zoom at calculated max Speed (km h^−1^) on streets.

The spatial distribution of the speeds for each vehicle are shown in Fig. 3a and c and associated with each street on Fig. 3b and d. Motorways reach higher speeds with values between 88 and 110 km h^−1^ shown in black. At major streets near the cities the average speeds oscillate between 44 and 88 km h^−1^ shown in orange. The lower speeds are found in the center of the cities shown in yellow.

**Fig. 4:**
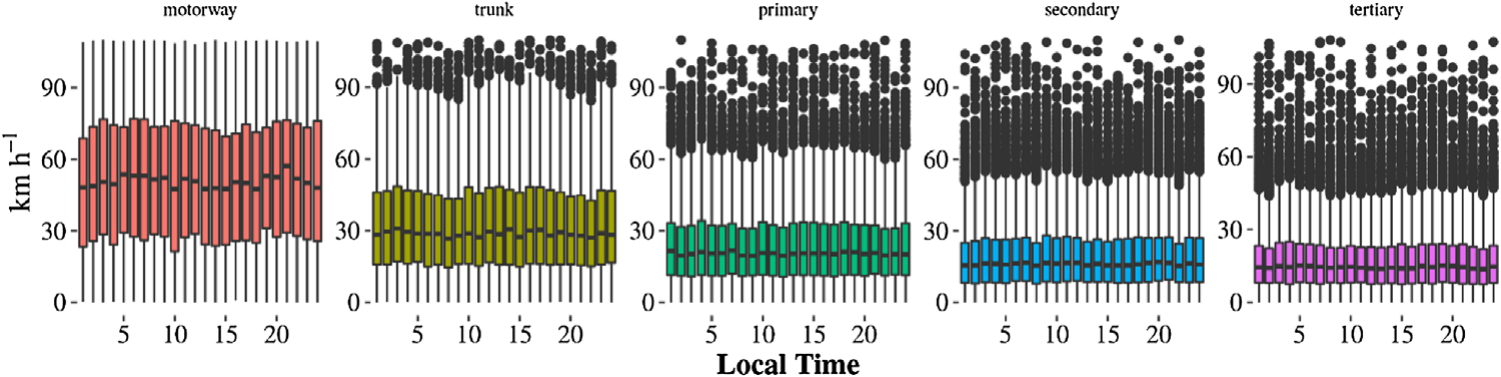
Temporal distribution of average max speed of Taxi (km h^−1^) by type of street.

The temporal distribution of speeds by type of street for the 24 h on Monday 2014-10-06 is shown on Fig. 4. Higher speeds are found in motorways and the speeds diminish through the hierarchy of the type of street from motorway to tertiary which seems reasonable. The interquartile range of speeds also lowers with descending street type from motorway to tertiary. Regarding the hourly distribution, motorways exhibited the most distinct temporal distributions with speeds increasing in the early hours and decreasing during morning and evening rush-hours, then increasing again into the evening. Primary, secondary and tertiary do not present a noticeable variation at different hours.

As already mentioned, GPS coverage is not uniform. Commercial fleets like Taxis and Trucks have a higher proportion of GPS coverages than the private Car fleet. Similarly, data for the different vehicle types is differently distributed geographically, by road type and by operating hours (almost likely as functions of fleet coverage, and operating practices). As a result, directly extrapolating these initial measurements of vehicle speed to larger local fleet would, most probably, introduce significant bias. This effect is likely to be most pronounced for Cars because the spatial behavior of Trucks is likely to be more regular because it often involves a high proportion of similar journeys (e.g. deliveries on fixed routes) and both Taxis and Trucks GPS coverage is likely to be higher because efficient navigation and therefore on-board GPS are common tools in both commercial practices. One way to minimize this bias is to use the speed as proxy of traffic flow. In this way, it is less crucial to have GPS for all streets monitored, but it is still important to consider local coverage.

### Fill

Some Open street Map roads do not have the number of lanes identified, and were hole-filled using average by type of street.

#### Flow

We compared GPS data with that from a data-base of simultaneous speed and traffic flow measurements made by the Traffic Engineering Company of São Paulo (CET, 2013) to investigate potential bias. These traffic count measurements are publicly available on http://www.cetsp.com.br/sobre-a-cet/relatorios-corporativos.aspx and they provide measurements of traffic flow and speed for morning and evening rush hours. Vehicle types reported are LDV, Motorcycles, Trucks with 2, 3 and 4 axes, and Urban and Rented Buses. The periods of time with measurements are 07:00-08:00, 08:00-09:00, 09:00-10:00, 17:00-18:00, 18:00-19:00 and 19:00-20:00 LT and they are spatially located inside the city focused on the main routes with the objective of characterizing the principal traffic flows in the area. Ibarra-Espinosa et al. (2017) geo-referenced the data for the year 2012 over an OpenStreetMap road network, uploaded into Mendeley Data web services, doi: 10.17632/rz2cymv6c2.1, and we used this data to correct the spatial bias. The procedure involved the calculation of ratios of local traffic flow of LDV and Trucks and speed for each street type and number of lanes. The GPS-derived speeds of LDV and Trucks were multiplied by these ratios for all street types and number of lanes. In this way, a GPS traffic flow proxy is derived from average speed GPS data, and the traffic flow / speed trends observed for each street type / lane number combination. The formula for this procedure is shown in Eq. (1).

(1) FC_ts,nl_ = SGPS_ts,nl_ * (FR_ts,nl_ / SR_ts,nl_)

Where FC_ts,nl_ is the traffic flow corrected for the type of street ts, number of lanes nl. SGPS_ts,nl_ is the average speed obtained from the GPS recordings at streets indicating the type and number of lanes. FR_ts,nl_ and SR_ts,nl_ are the Traffic flow and Speed recorded during CET measurements made between 08:00 and 09:00 in 2012. There are no traffic counts for Motorways, therefore we assigned the same values of Trunks to Motorways. Table 2 summarizes this work and shows the tendency for speeds and flows to both increase with street type hierarchy, tertiary to motorway, and lane number. However, the speed does not increase linearly. There are also small junction streets designated as 'link' in the data base, e.g. motorway_link. In OpenStreetMaps, ‘links’ are small roads or ramps that connect roads of different hierarchy, for example, small rings in motorways used to carry the traffic from one type of street to another (http://wiki.openstreetmap.org/wiki/Highway_link). The traffic corrections in these streets were divided by two, to smooth flows through this change-point.

**Table 2:** Average traffic and speeds of Cars by type of street and number of lanes at 08:00-09:00 in São Paulo, 2012.

Type of street	Lanes	Cars veh h^−1^	Speed km h^−1^	Cars / Speed
Motorway and Trunk	>= 10	9615.50	69.15	139.05
Motorway and Trunk	< 10 & >= 8	4709.07	60.62	77.68
Motorway and Trunk	< 8 &>= 6	4416.85	54.01	81.78
Motorway and Trunk	< 6 & >= 4	3282.50	51.53	63.70
Motorway and Trunk	< 4	786.50	16.00	49.16
Primary	>= 8	4486.50	61.69	72.73
Primary	= 7	2664.00	30.80	86.49
Primary	= 6	3233.25	37.30	86.67
Primary	< 6 & >= 4	3311.20	35.88	86.67
Primary	< 4	2697.00	29.50	91.42
Secondary	>= 8	1671.00	26.00	64.30
Secondary	< 8 & >= 6	2388.00	36.64	65.17
Secondary	= 5	1295.00	28.90	44.81
Secondary	= 4	2527.00	26.43	95.60
Secondary	< 4	626.50	27.50	22.78
Tertiary	>= 4	1600.00	22.00	72.73
Tertiary	< 4	804.00	15.90	50.57

Only 8% of the streets strictly fulfill the criteria for this correction. So, we calculated the average number of lanes by type of street for the remainder and filled the gaps where there was no information of number of lanes. To calculate these averages, we used the same data used to make Table 2. The rounded average number of lanes per street were: motorway 3, motorway_link 1, trunk 3, trunk_link 2, primary 3, primary_link,2, secondary 2, secondary_link 1, tertiary 2 and tertiary_link 1. When comparing this information with the data from Table 2, it should be noted that we are working with the OpenStreetMap road network for São Paulo and the characteristics might not be readily applied to other regions. Therefore, it is possible that the number of vehicles and speed per type of street and number of lanes, and the average number of lanes per type of street, be different from those that we used in the present work.

The specification presented are meant for traffic flow generated from GPS recordings of Cars. In the case of Trucks, we only corrected the traffic circulating in motorways and trunks using the ratio of the average number of trucks in trunk streets, 101.06 veh h^−1^ and the average speed for the same type of streets, 50.26 km h^−1^. The correction ratio obtained (2.01) was then applied to truck flows on motorway and trunk roads.

**Fig. 5:**
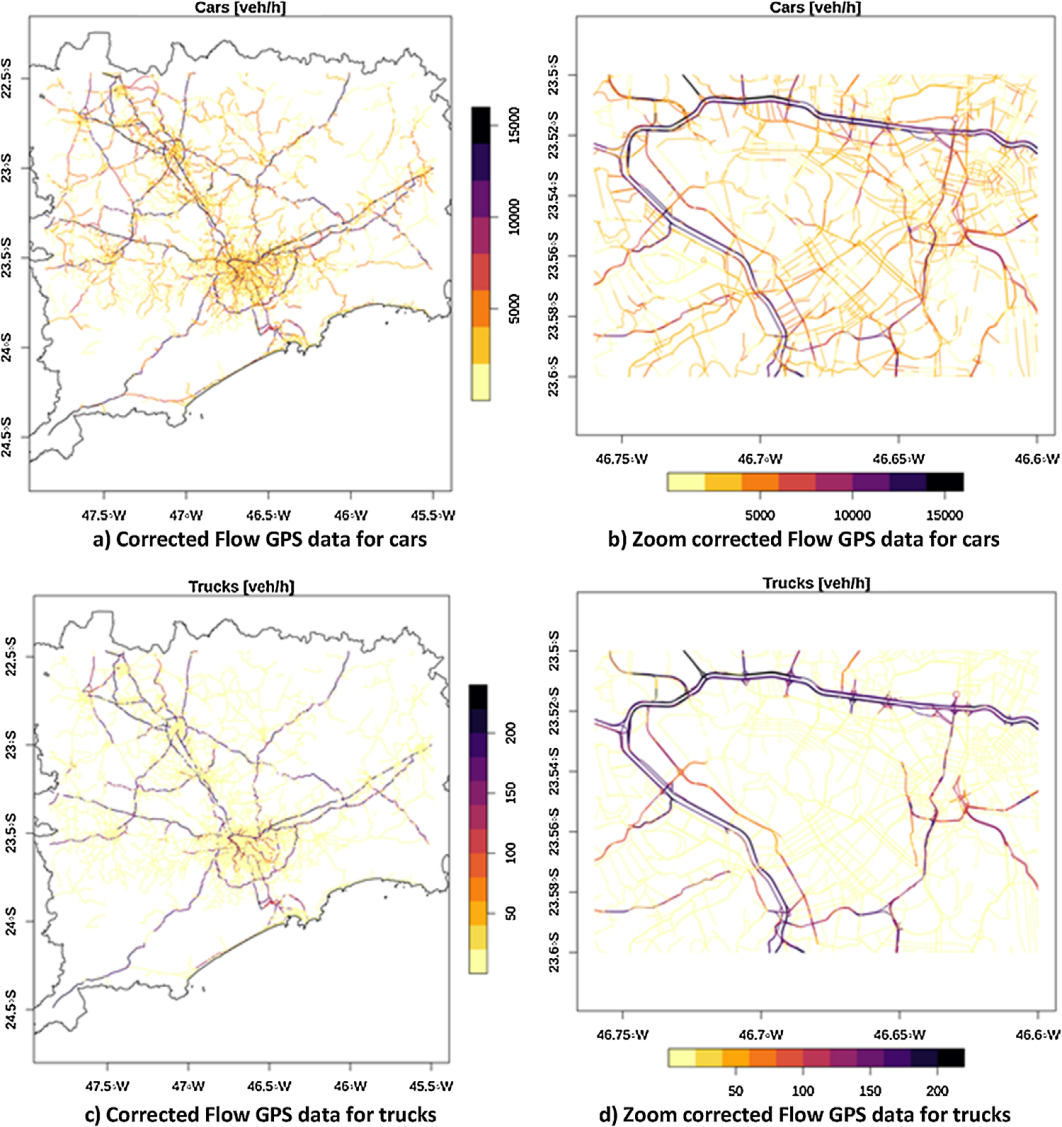
Traffic flows derived from GPS recordings for Cars a), for Cars with zoom over São Paulo, Trucks c) and Trucks with zoom over São Paulo.

The GPS recordings are showing the spatial bias are shown in Fig. 2b, c and d. The spatial bias of this data was driven by a higher density of vehicles in these areas and this was simply because there were more vehicles providing GPS data operating in this part of the city. However, when we estimated the flow from speeds recordings according the criteria shown in Table 2, we saw an improvement in the traffic flow as shown in Fig. 5. Now, the traffic volume exceeds 15,000 veh h^−1^. Also, the traffic is densest in urban motorways near the center of the city. Although we cannot be certain that all bias was removed, the resulting spatial distribution of both traffic and the number of vehicles per lane seemed reasonable throughout the network.

We presented these aspects in Figs. 2c, 3 d, 5 b and d for the center of the city of São Paulo to highlight both the bias in speed and flow estimates derived from current GPS data sources and the effectiveness of this strategy to develop and apply corrections across large areas. The resulting traffic flow profile for the region is shown in Fig. 5a and b for Cars and Fig. 5c and d for Trucks. The resulting traffic flow of Cars is concentrated in main roads near the center of each region. For instance, Car traffic densities are highest in city center of Campinas, São Paulo and Santos. There are streets where Car traffic exceeds 15,000 veh h^−1^. However, the total amount of Cars is 18,413,811 veh h^−1^ and as the number of streets with Cars is 8,459, there are on average 2176.83 veh h^−1^ street^−1^. In the case of Trucks, they are concentrated on the motorways. Truck volumes can exceed 200 veh h^−1^ in the most congested streets which seems a low value. However, the total amount of Trucks is 296,584.4 veh h^−1^ and as the number of streets with Trucks is 2,774, the average number per street is 106.92 veh h^−1^ street^−1^. It is important to remember that traffic flow has higher density on few major roads, and most of the roads has a very small number of trucks on circulation.

## Comparison

Nyhan et al. [7] have also presented a work where GPS data was used to estimate traffic information as inputs for a regional emission model. Table 3 shows a comparison of Nyhan et al. [7] and this study. The number of GPS recordings and the temporal coverage are similar, but vehicle types are different. The GPS data used by Nyhan et al. [7] was derived purely from Taxis, whilst our data source incorporated several vehicle types. The spatial area covered in our study is also 88 times bigger than that in Nyhan et al. [7]. The method for generating traffic flow was neural networks [8], whilst we used an admittedly cruder but less computer-intensive and widely applicable assignment strategy. Although speed as a proxy for interpolating traffic data is not fool-proof, we believe that using corrections based on the proportion of observed traffic counts and speeds minimize these possible errors and this suggestion is corroborated by realistic traffic flow outputs as shown in Fig. 5.

**Table 3:** Comparison of data and methods between Nyhan et al [7] and this study.

	Nyhan et al [7]	This study
Number of GPS recordings and type of vehicles	120 million from Taxis	124 million from Cars, Trucks and Taxis and Undefined
Spatial coverage	Singapore island (722 km^2^)	Metropolitan areas of São Paulo, Santos, Sorocaba, São José dos Campos and Campinas (64,080 km^2^)
Timespan	From 21-02-2011 to 27-02-2011	From 04-10-2014 to 10-10-2014
Filter criteria	Speed: 150 km h^−1^, acceleration: 10 m s^-2^	Speed: 110 km h^−1^, acceleration: 10 m s^-2^
Consecutive recordings	5 seconds	Average of 11 minutes
Method for extrapolating traffic	Neural network [8]	Proportion observed Speed and traffic count by type of street and number of lanes

## Validation and future refinement

The traffic flows generated using this method were validated by comparing the mean and standard deviation of vehicles by type of street between GPS traffic flow and the traffic counts data-base [9], as shows Table 4. The mean values show good agreement for most of groups, however, the GPS mean traffic on Trunk streets is higher than the counts. We think that this is an artefact of current methods and data sources. Traffic count data is mostly collected in the São Paulo city-center where there is less Truck activity and lower speeds. By comparison, the GPS data covers a much larger area. Our current method extrapolates counts across this larger area, which is an obvious compromise in the absence of more complete information in areas outside the city-center and for some road type / lane number combinations. Our recommendation, moving forward, is that future studies, wherever possible, use traffic count data to more fully characterize issues associated with multi-lane and in- and out-of-city-center coverage. Nevertheless, the GPS mean values are increase incrementally with the hierarchy of the type of street, which is expected. The mean values for the traffic count also show this tendency, except for the tertiary roads, for which the average number is higher than for secondary streets. However, this could be related to the small sample size (n = 3 for counts on tertiary streets). Also, the traffic counted on tertiary roads often overlaps with that on secondary roads, i.e., these are sometimes the same streets with different categorization. Finally, we conclude that the traffic flow information generated from GPS data can reproduce the general aspects of traffic count by type of street and is suitable for input in a bottom-up vehicular emissions inventory if sampling bias is corrected robustly.

**Table 4:** Statistics of GPS traffic flow 2014 and traffic counts 2012 in São Paulo at 08:00-09:00.

Type of street	GPS Mean	GPS Standard deviation	GPS n	Count Mean	Count Standard deviation	Count n
Trunk	4001.05	3290.38	1784	2857.03	1783.20	94
Primary	2036.57	1713.53	4607	2077.04	840.71	85
Secondary	1745.58	1606.49	7184	1208.29	664.74	24
Tertiary	1617.40	1619.23	7556	1324.67	447.10	3

## References

[1] Amini S., Gerostathopoulos I., Prehofer C. (2017). Big data analytics architecture for real-time traffic control. Models and Technologies for Intelligent Transportation Systems (MT-ITS), 2017 5th IEEE International Conference on.

[2] Hoffman A.J., Venter W.C. (2017). Application of data analytics to transport corridor diagnostics and performance benchmarking. Intelligent Transportation Systems (ITSC), 2017 IEEE 20th International Conference on (pp. 1-8).

[3] Stipancic J., Miranda-Moreno L., Saunier N. (2017). Impact of congestion and traffic flow on crash frequency and severity: application of smartphone-collected GPS travel data. Transp. Res. Record: J. Transp. Res. Board.

[4] Ibarra-Espinosa S., Ynoue R., O’Sullivan S., Pebesma E., Andrade M.D.F., Osses M. (2018). VEIN v0.2.2: an R package for bottom–up vehicular emissions inventories. Geosci. Model. Dev. Discuss..

[5] Dowle Matt, Srinivasan Arun (2018). data.table: Extension of `data.frame`. R Package Version 1.11.8. https://CRAN.R-project.org/package=data.table.

[6] Pebesma E. (2018). Simple features for r: standardized support for spatial vector data. R J..

[7] Nyhan Marguerite, Sobolevsky Stanislav, Kang Chaogui, Robinson Prudence, Corti Andrea, Szell Michael, Streets David, Lu Zifeng, Britter Rex, Barrett Steven R.H., Ratti Carlo (2016). Predicting Vehicular Emissions in High Spatial Resolution Using Pervasively Measured Transportation Data and Microscopic Emissions Model. United States: N. P..

[8] Moretti F., Pizzuti S., Panzieri S., Annunziato M. (2015). Urban traffic flow forecasting through statistical and neural network bagging ensemble hybrid modeling. Neurocomputing.

[9] Ibarra-Espinosa S. (2017). Traffic counts both ways cet 2012 sao paulo. Tech. rep., Medeley Data.

